# Peroxymonosulfate- and Persulfate-Based Advanced Oxidation Processes for Soil Remediation: A Review

**DOI:** 10.3390/molecules31193553

**Published:** 2026-10-06

**Authors:** Antonio Iorio, Aqsa Nawaz, Elio Coppola, Stefano Salvestrini

**Affiliations:** 1 Department of Engineering, University of Campania “Luigi Vanvitelli”, Via Roma 29, 81031 Aversa, Italy; antonio.iorio1@unicampania.it (A.I.); aqsa.nawaz@unicampania.it (A.N.); 2 Department of Environmental, Biological and Pharmaceutical Sciences and Technologies, University of Campania “Luigi Vanvitelli”, Via Vivaldi 43, 81100 Caserta, Italy; elio.coppola@unicampania.it

**Keywords:** peroxymonosulfate, persulfate, peroxydisulfate, advanced oxidation processes, AOPs, soil remediation, soil decontamination, soil depollution, soil organic pollutants

## Abstract

The presence of organic pollutants in soil represents one of the main remediation challenges of the 21st century, and in this regard, peroxymonosulfate- (PMS) and persulfate (PS)-based advanced oxidation processes (AOPs) are among the most promising technologies for in situ contaminant removal. However, the complex and heterogeneous nature of soil strongly influences (positively or negatively) the efficacy of PMS/PS-AOPs, necessitating the development of better-targeted application strategies. This review provides a comprehensive overview of state-of-the-art applications, mechanistic pathways, operational limitations, and future perspectives of PMS/PS-based treatments for soil remediation. Self- and external activation methods of PMS and PS in soil, including thermal activation, microwave field stimulation, ultrasound cavitation, transition metal catalysis, alkaline activation, mechanochemical ball milling, and electrokinetic transport system, are evaluated. A survey of the recent literature reveals laboratory-scale tests to have excellent efficiency in removing various low-molecular-weight organic pollutants, allowing the research to focus toward more complex molecules. However, large-scale application remains constrained by local soil characteristics, high operational costs, and the adverse impacts of such treatments on the soil biotic community and fertility. The synergistic combination of multiple techniques is emerging as an effective strategy to bridge the efficiency gap between liquid and solid matrices. This approach could mitigate adverse environmental side effects and optimize low-invasiveness treatments for large-scale soil remediation.

## 1. Introduction

The release of toxic organic substances from various human activities into soil is one of the main topics in scientific discussions. The high resistance to natural degradation processes permits these pollutants to persist for a long time, causing damage to ecosystems and human health [1,2,3,4]. The filtering property of soil prevents them from reaching the underlying aquifer too rapidly, acting as a protective barrier between underground water sources and contaminants from the surface. On the other hand, the excessive accumulation of these substances damages its chemical and physical properties [5]. More than three-quarters of European agricultural soils contain detectable quantities of pesticide active ingredients (both authorized and banned) and/or their degradation products [6]. The occurrence of organic contaminants is on an increasing trend, underlining the strong need for regulatory policies to safeguard soil as well as the underlying aquifers [7]. Due to their tendency to be strongly sorbed into the soil’s organic/inorganic colloids (humus/clay phyllosilicates), organic pollutants are generally resistant to traditional remediation techniques [8]. The application of new technologies, such as advanced oxidation treatments, may favor the degradation of polluting molecules, thus providing greater protection for soil and aquifers [9].

Advanced oxidation processes (AOPs) are a set of techniques aimed at the partial or complete chemical oxidation of organic molecules by means of reactive oxygen species (ROS) or other reactive substances (e.g., metal elements that exhibit multiple oxidation states, such as iron and manganese), via radical and non-radical pathways. ROS include hydroxyl radical (HO^•^), sulfate radical (SO_4_^•−^), superoxide radical (O_2_^•−^) and singlet oxygen (^1^O_2_). They are generated during AOPs by the activation of strong oxidizing agents such as hydrogen peroxide (H_2_O_2_), peroxymonosulfate (PMS, HSO_5_^−^), peroxydisulfate or more commonly known as persulfate (PS, S_2_O_8_^2−^), and ozone (O_3_). The activation of these oxidants can occur in different ways, including by means of heat, homogeneous/heterogeneous catalysis (e.g., using transition metal-based catalysts), electrochemical treatment and photocatalysis. When applied to soil, AOPs can oxidize the organic pollutants up to complete mineralization or transform them into products susceptible to biodegradation processes. Among AOPs, Fenton-based processes have been widely studied. The traditional Fenton reaction (homogeneous Fenton oxidation) uses hydrogen peroxide and dissolved iron species (e.g., Fe^2+^) to generate hydroxyl radicals that degrade the pollutants. The process can take place at environmental temperature and atmospheric pressure, but it needs to maintain a low pH range (2.5–4.0) to prevent the precipitation of iron, thus limiting its application to the soil [2].

(1) $${Fe}^{2+}+H_{2}O_{2}\to {Fe}^{3+}+HO^{•}+{HO}^{-}$$

Alternative Fenton processes make use of solid Fe-based catalysts (heterogeneous Fenton-like processes). They are particularly attractive for soil remediation showing more potential efficacy and applicability than traditional Fenton process, although stability, longevity and non-toxicity of the catalysts remain a major concern [10]. More recently, PMS/PS-based AOPs have attracted the attention of researchers because they use sulfate radicals for pollutant oxidation. Sulfate radicals can be preferred over hydroxyl radicals for different reasons, including higher redox potential, longer half-life, and wider pH range applicability [11]. Moreover, the activation of PS and PMS can generate reactive oxygen species other than sulfate radicals, including hydroxyl radicals and singlet oxygen. In this review, the PMS/PS-based AOPs for soil remediation are examined. The aim is to give an overview of the state of the art of their applications in soil, their limitations and future directions.

## 2. PMS/PS-AOPs

**Figure 1:**
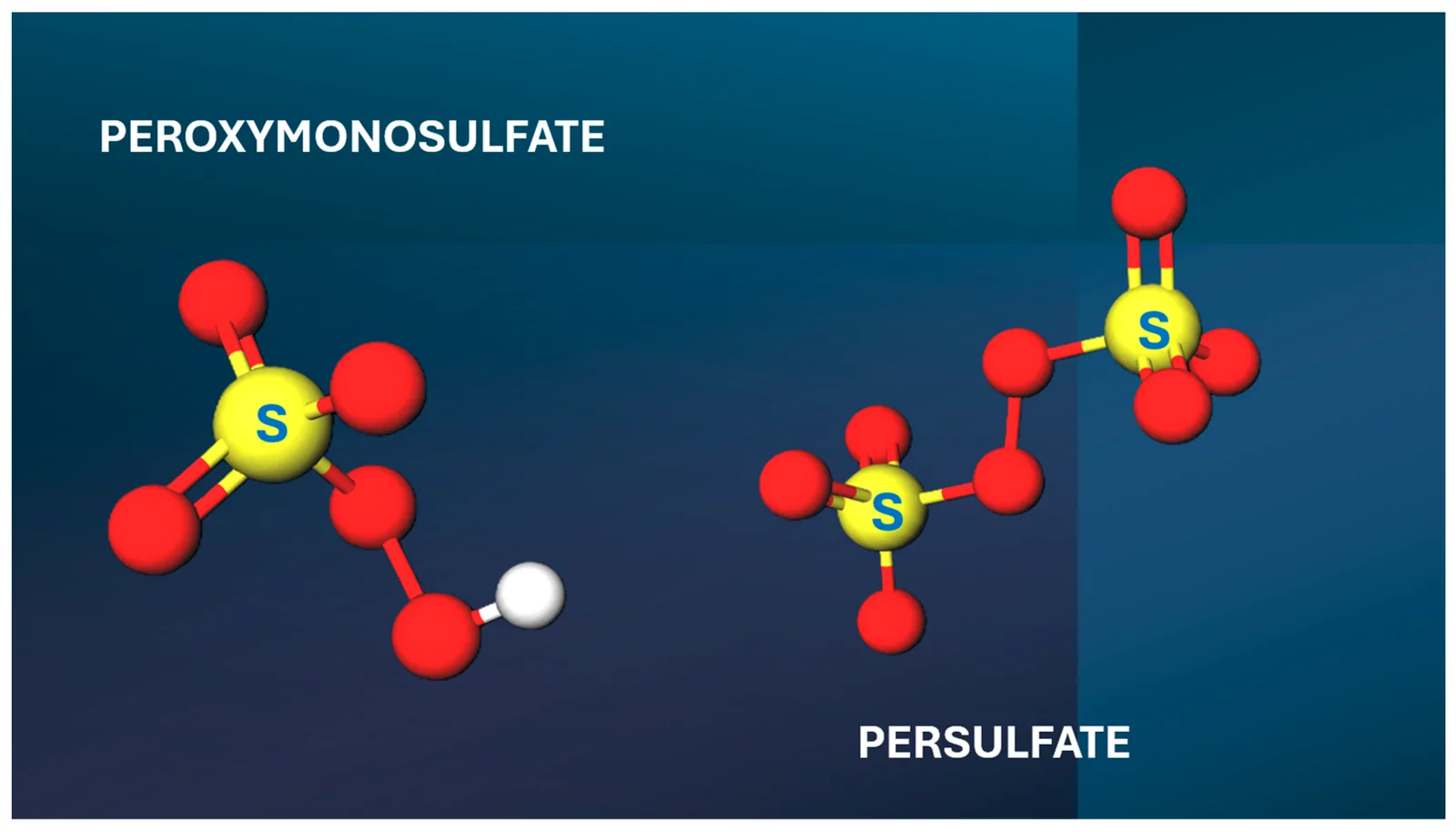
Chemical structure of PMS and PS.

Sulfate-radical AOP technology has been widely studied in recent years for the treatment of organic pollutants in soils [2]. The reasons behind the great interest in the use of the sulfate radical are to be found in its superior performance for the abatement of organic pollutants compared to hydroxyl radicals. In fact, SO_4_^•−^ has longer half-life (~10^−6^ s of SO_4_^•−^ vs. ~10^−9^ s of HO^•^), higher selectivity toward some organic pollutants (e.g., electron-rich compounds like phenols and anilines), and a higher oxidation potential (2.5–3.1 V vs. 1.8–2.7 V) [8,12]. Sulfate radicals are generated from the activation of PS and PMS. PS and PMS are two inorganic anions characterized by the presence of a peroxide bond that links together two sulfate groups in PS, and a sulfate group with a hydroxyl group in PMS (see Figure 1).

Depending on the specific activation mechanism involved, e.g., energy transfer or electron transfer, one or two sulfate radicals can be formed through the peroxide bond cleavage of PMS/PS. Moreover, other two main ROS (hydroxyl radical and singlet oxygen) can be generated via additional reaction pathways. For example, sulfate radicals can be converted into hydroxyl radicals under neutral or alkaline conditions or into persulfate radical (S_2_O_8_^•−^) that in turn may react to give singlet oxygen [2,13].

(2) $${S_{2}O_{8}}^{2-}\overset{Energy}{\to }{SO}_{4}^{•-}+{SO}_{4}^{2-}$$

(3) $${S_{2}O_{8}}^{2-}+M^{n+}\to {M^{\left(n+1\right)+}+SO}_{4}^{•-}+{SO}_{4}^{2-}$$

(4) $${S_{2}O_{8}}^{2-}+{SO}_{4}^{•-}\to {SO}_{4}^{2-}+{S_{2}O_{8}}^{•-}$$

(5) S2O8•−+H2O→SO4•−+HSO4−+H++1/2 O21

(6) $${HSO_{5}}^{-}\overset{Energy}{\to }{SO}_{4}^{•-}+HO^{•}$$

(7) $${HSO_{5}}^{-}+M^{n+}\to M^{\left(n+1\right)+}+{SO}_{4}^{•-}+HO^{-}$$

(8) HSO5−+SO52−→HSO4−+SO42−+O21

(9) $${SO}_{4}^{•-}+H_{2}O\to {SO}_{4}^{2-}+HO^{•}+H^{+}$$

(10) $${SO}_{4}^{•-}+HO^{-}\to {SO}_{4}^{2-}+HO^{•}$$

In the above equations, M represents a generic redox species.

PS is the more widely used oxidant of the two; it has a longer half-life, lower cost and higher oxidation potential [1,2]. On the other hand, PMS requires less energy to generate sulfate radicals. PMS salt is available in potassium form (KHSO_5_), whereas PS can be found as sodium (Na_2_S_2_O_8_), potassium (K_2_S_2_O_8_) and ammonium ((NH_4_)_2_S_2_O_8_) salt. K_2_S_2_O_8_ is the least used due to its low solubility (53 g L^−1^ at 20 °C), compared to sodium and ammonium salts (556 g L^−1^ and 582 g L^−1^ at 20 °C, respectively). Low cost and major stability make Na_2_S_2_O_8_ the most utilized salt. The use of the ammonium salt has the advantage of increasing the bioavailability of nitrogen in soil, favoring the post-treatment recovery of bacterial community, but with the drawback of suppressing sulfate radical activity and thus reducing the pollutants removal efficiency [1,2,14,15].

One of the most important issues in the application of PMS and PS for soil remediation is the introduction of an excess of sulfate ions and radicals into the matrix [16], which leads to a decrease in pH and thus to a depletion of the soil bacterial load. The detrimental effects of generated acidity can also be extended to plant organisms, although they are only temporary and resolved within a few weeks [17]. Among the solutions proposed to overcome this problem, there is the use of additional reagents that do not act at excessively acidic pH and that limit the PMS/PS dosage, such as permanganate [13,18]. Alternatively, the combined use of multiple AOP technologies is recommended, also to exploit their synergistic effects [1,16].

The use of PMS/PS in soil has been shown to be effective on numerous species of polycyclic aromatic hydrocarbons (PAHs) [16,17,19]. The reaction kinetics of PMS/PS-AOPs in soils is strongly influenced by the local soil characteristics. Aspects such as permeability, composition of the mineral fraction and the quantity of soil organic matter actively influence, positively or negatively, the effectiveness of the treatment. For example, it has been observed in some cases that high soil organic matter content significantly slows down the rate of pollutant mineralization. Another aspect to be considered is the possible formation of toxic intermediates, resulting from the interaction of persulfate with soil organic matter [16,20]. The concentration of pollutant into the soil is another important factor to be considered for evaluating the applicability of the PMS/PS treatment. According to Lemaire et al., the use of PS is not recommended in lowly contaminated soils [21]. In such conditions, the limited pollutant degradation benefits of PS are overcome by the negative effects on the soil’s bacterial community owing to excessive lowering of the pH. To treat soils with low amounts of pollutants, other oxidants such as permanganate and Fenton reagents, should be preferred.

## 3. Effect of Soil Physicochemical Characteristics on the PMS/PS Effectiveness

PMS and PS are very weak oxidants in their inactivated form and hardly decompose spontaneously at room temperature. Despite this, both can oxidize organic contaminants in the soil thanks to the presence of natural constituents capable of promoting the formation of ROS, even without the addition of external activators. This is particularly true for PMS that is less stable than PS and thus requires a lower energy input to be activated [22]. On the other hand, a high content of soil constituents, e.g., organic matter or dissolved anions, can have the opposite effect, suppressing the activity of the oxidants. Soil aging is another crucial aspect to consider in oxidant performance.

### 3.1. Mineralogical Composition

The mineralogical composition of the soil can have a major impact on the degradation process of PMS/PS in the presence of elements such as Mn and Fe, especially in soils with low organic matter content [23,24]. According to Peng et al. [25], the decomposition of PS by soil minerals occurs via two pathways, one radical and one non-radical. In the non-radical pathway, PS is decomposed to SO_4_^2−^ on the mineral surface with the reduction of the active metal sites (e.g., Mn and Fe). During the radical reaction pathway, PS oxidizes the mineral active sites and produces SO_4_^•−^ and HO^•^ that are responsible for the pollutant degradation. Radical and non-radical pathways were reported also by Liang et al. for the PS oxidation of sulfamethoxazole [26] in Fe-poor and Fe-rich soils. The authors found that in Fe-poor soils, the classical radical pathway generating SO_4_^•−^ and HO^•^ is dominant. In Fe-rich soils (e.g., in the presence of minerals like ilmenite), a non-radical mechanism takes place without requiring the formation and diffusion of reactive oxidizing species in the aqueous phase; that is, by a surface-confined electron transfer: (i) PS forms a complex with the Fe mineral; (ii) the complexed PS exhibiting a high redox activity accepts electrons efficiently from the sorbed contaminant which is thus oxidized.

(11) $$Fe\left(II\right)mineral+PS\to Fe\left(II\right)mineral-{PS}^{*}$$

(12) $$Fe\left(II\right)mineral-{PS}^{*}+C{ontaminant}_{\left(sorbed\right)}\to {Contaminant}_{\left(ox\right)}+Fe\left(II\right)mineral+2SO_{4}^{2-}$$

The presence of Fe in soil was found to be crucial also in the work by Chen and co-workers for the efficient activation of PMS [27]. According to the authors, Fe oxides favor a non-radical pathway generating ^1^O_2_:

(13) $$\equiv {Fe}^{3+}-OH^{-}+HSO_{5}^{-}\to \equiv {Fe}^{2+}-SO_{5}^{•-}+H_{2}O$$

(14) 2≡Fe2+−SO5•−+2H2O→2≡Fe3+−OH−+O21+2SO42−+2H+

### 3.2. Organic Matter Content

As regards soil organic matter (SOM), its amount and chemical characteristics can strongly affect, both positively and negatively, the activation and efficacy of PMS/PS oxidation [23]. SOM behaves as a redox-active substrate capable of modifying the nature and availability of reactive species. For example, some organic soil constituents are capable of positively interacting with both PS and PMS via electron transfer. More specifically, the phenolic and quinone-type structures of soil organic matter generate low-molecular-weight alkyl fragments (RHs). When RHs react with sulfate radicals and hydroxyl radicals, R^•^ radicals are formed. The R^•^ radicals can then react with PS, breaking the O–O bond by electron transfer. As SOM increases, the predominant process switches from an oxidative degradation system, driven by SO_4_^•−^ and HO^•^, to a reductive degradation system, driven by R^•^ [23,28]. The pivotal role of phenolic and quinonic groups of SOM in the activation of PMS and PS can also occur through other mechanisms. For example, deprotonated phenols can activate PS via electron transfer of one electron generating sulfate radicals [29].

(15) $$-ArO^{-}+S_{2}O_{8}^{2-}\to SO_{4}^{2-}+SO_{4}^{•-}+-ArO_{ox}$$

Other studies report a positive synergy between low SOM and Fe (II)/Fe (III) concentration. Fe (II) acts as PS activator as in a classical Fenton-like reaction, producing sulfate radicals as dominant ROS and Fe (III). The role of SOM functional groups –OH and –COOH is to chelate iron atoms and promote the regeneration cycle between its two oxidation states [30]. The low frequency of interaction between sulfate radicals and SOM is an important factor for soil application, due to the competitive effect of sulfate radicals with the organic pollutant they are intended to remove. Fang et al. report that the undesired consumption of radicals is not relevant in soil with low organic matter content [23]. In the presence of a higher amount of SOM, the suppression of sulfate (and hydroxyl radicals) can be overcome by the generation of singlet oxygen from PMS according to Equation (8) [31].

Unlike the radical species produced, singlet oxygen acts more selectively toward target pollutants, being less susceptible to the SOM scavenging effect. Delocalized π-electrons (C–π) of condensed aromatic rings, quinones, and conjugated double bonds in SOM can be involved in the PMS activation and singlet oxygen production through the following electron transfer mechanism mediated by peroxymonosulfate radical (SO_5_^•−^) [32]:

(16) $$HSO_{5}^{-}+C-\pi \to SO_{4}^{•-}+HO^{-}+C-\pi^{+}$$

(17) $$HSO_{5}^{-}+C-\pi^{+}\to SO_{5}^{•-}+H^{+}+C-\pi$$

(18) 2SO5•−→2SO42−+O21

The formation of singlet oxygen is documented also in the presence of 1,4-benzoquinone (BQ). BQ can be found in soil as an oxidation intermediate product of several small dissolved organic compounds, including phenolic and benzoic derivatives. In the literature, it is suggested that BQ can act as a catalyst for the activation of PMS by forming a BQ/PMS intermediate (1,4-benzoquinone bis-dioxirane) which subsequently rapidly reacts with SO_5_^2−^ to produce ^1^O_2_ [33,34]. Similarly to soil organic matter, dissolved soil organic matter can affect the efficacy of the oxidant treatment. Shang et al. showed that ^1^O_2_ generated by microwave-activated PMS treatment preferentially reacts with dissolved organic matter rich in N [35].

### 3.3. Dissolved Anions

The ionic composition of the soil liquid phase is another key factor controlling the effectiveness of PMS/PS-based AOPs, as some inorganic anions can compete with the contaminants for the radical species generated during oxidant activation. The most common anions present in the soil liquid phase include SO_4_^2−^, Cl^−^, HCO_3_^−^/CO_3_^2−^, NO_3_^−^ and to a lesser extent NO_2_^−^ [36]. Unlike SO_4_^2−^ and NO_3_^−^, the other anions^−^ exert a scavenging effect on SO_4_^•−^ and HO^•^ whose strength strongly depends on the relative anion concentration and on the amount of oxidants used in the process [37,38].

Chloride ion (Cl^−^) is a strong scavenger. The consumption of sulfate and hydroxyl radicals by Cl^−^ leads to the production of the less reactive Cl^•^ radical [39].

(19) $${Cl}^{-}+SO_{4}^{•-}⇌SO_{4}^{2-}+{Cl}^{•}$$

(20) $${Cl}^{-}+HO^{•}\to HO^{-}+{Cl}^{•}$$

Bicarbonate (HCO_3_^−^) and carbonate (CO_3_^2−^) are moderate to strong scavengers, able to suppress SO_4_^•−^ and HO^•^ activity according to the following reactions:

(21) $$HO^{•}+HCO_{3}^{-}\to CO_{3}^{•-}+H_{2}O$$

(22) $$HO^{•}+CO_{3}^{2-}\to CO_{3}^{•-}+HO^{-}$$

(23) $$SO_{4}^{•-}+HCO_{3}^{-}\to CO_{3}^{•-}+HSO_{4}^{-}$$

(24) $$SO_{4}^{•-}+CO_{3}^{2-}\to CO_{3}^{•-}+SO_{4}^{2-}$$

CO_3_^2−^ is a stronger inhibitor than HCO_3_^−^ (especially toward hydroxyl radicals), resulting in a more pronounced scavenging at alkaline pH (where CO_3_^2−^ dominates) [40]. The formed carbonate radical (CO_3_^•−^) exhibits a moderate-to-weak oxidant strength as compared to SO_4_^•−^ and HO^•^.

Nitrite anion (NO_2_^−^) deserves a separate discussion. Its presence in the soil solution must be carefully taken into consideration during AOPs due to the possible formation of hazardous substances. NO_2_^−^ can significantly decrease the oxidation activity of both SO_4_^•−^ and HO^•^ (see Equations (25) and (26)). In addition, the scavenging reactions generate the nitrogen dioxide radical (^•^NO_2_) that can react with phenols to produce harmful nitroaromatic compounds [41].

(25) SO4•−+NO2−→NO2•+SO42−

(26) HO•+NO2−→NO2•+HO−

### 3.4. Soil Aging

Soil aging represents one of the major factors controlling the efficiency of PMS/PS-based AOPs in the remediation of contaminated soils. Soil aging is a long contaminant–soil contact that causes a progressive redistribution of the contaminant within less accessible areas of the soil matrix, involving stronger sorption to soil organic matter and mineral surfaces, diffusion into micropores and intra-aggregate domains, resulting in a slowed degradation kinetics and in the formation of increasingly sequestered pollutant fractions with reduced desorbability. These factors lead to a progressive decrease in the amount of contaminant degradable by oxidants [42,43].

Zhang et al., for example, investigated the correlation between the PAH aging time and removal efficiency, confirming that pollutant degradation progressively decreases as residence time increases. Thus, aging shifts the controlling mechanism from predominantly reaction-limited oxidation toward a process increasingly influenced by contaminant accessibility and mass transfer [44]. The aging effect is also evident in petroleum-contaminated soils subjected to environmental weathering processes. Chen et al. specifically explored the PS oxidation of soils contaminated with aged lubricating oils, highlighting how such weathering promotes stronger sorption of hydrocarbons to the soil solid phase and their diffusion into soil pores, thereby linking aging to the contaminant’s availability for degradation [45]. A less marked aging effect was found by Gou et al. investigating the remediation of highly PAH-contaminated aged soil using PS. The authors reported substantial PAH removal, demonstrating that aged contaminants remain susceptible to PS oxidation, even though the removal strongly depended on PS dosage (negatively affecting bacterial abundance) and activation methods [17].

Thermal treatment can enhance the remediation of aged soils through a dual mechanism involving increased oxidant activation and enhanced contaminant accessibility (by widening pores and increasing the reactants ‘diffusion rate). On the other hand, heat can thermodynamically induce the desorption of contaminants sorbed via exothermic processes but it disfavors those sorbed via endothermic processes [46]. The actual effectiveness of this system varies depending on the nature of the molecule involved. This means that, for less volatile or strongly sequestered contaminants, a relatively mild heat treatment might have a limited effect [47,48,49]. However, excessive temperatures can also accelerate persulfate decomposition and non-productive oxidant consumption, thereby shortening the effective oxidation period. Consequently, the beneficial effect of thermal treatment depends on achieving a temperature range in which the increase in contaminant accessibility and reactive species generation outweighs the accelerated loss of oxidant [44].

## 4. Injection and Transport of PMS/PS in Soil

The introduction of PMS/PS into the soil takes place by injection of a concentrated aqueous solution of the oxidant using open-loop or recirculating systems. A spatial analysis of the contaminated area is necessary to choose the most appropriate injection system. For small areas, a direct injection system is generally used, while for large areas, an injection well with or without recirculation is preferred. The installation of permanent injection wells consists of the use of multiple vertical wells, obtained by drilling the soil up to the depth of the area to be treated. A matrix of wells guarantees the possibility of a more homogeneous coverage of the treatment and a greater dispersion of the oxidant in the affected area [50]. A more complex version involves the use of hydraulic circuits for the extraction of water from the underlying aquifer, mixing with the oxidant and reinjection into the subsoil. This system allows the creation of recirculation cells for better control of the injection flow and increasing the contact time between oxidant and contaminant. This methodology requires high volumes of solution, albeit at a lower concentration than other methods, and treatment durations ranging from 4 to 8 weeks [51]. In direct-push applications, probes capable of injecting the oxidizing solution at multiple time intervals are used. This method provides a temporary installation with a smaller injection radius than injection wells, but is therefore suitable for the treatment of localized areas or for attacking hot spots of contaminants in inhomogeneous soils. In this case, the treatment involves several injection cycles over 30–90 days [50,52].

An important aspect related to the transport of PMS/PS is the soil heterogeneity. Soil texture, structure, and composition change along its horizons affecting the movement of the oxidants. Fine-textured horizons are characterized by low permeability that hinder oxidant diffusion and strongly bind contaminants. A further obstacle is represented by the chemical composition of the substrate. For example, if the oxidant encounters a region rich in iron or organic matter along the way, it could be prematurely activated losing most of its oxidation capacity before reaching the polluted area [23,53].

### Electrokinetic Transport

**Figure 2:**
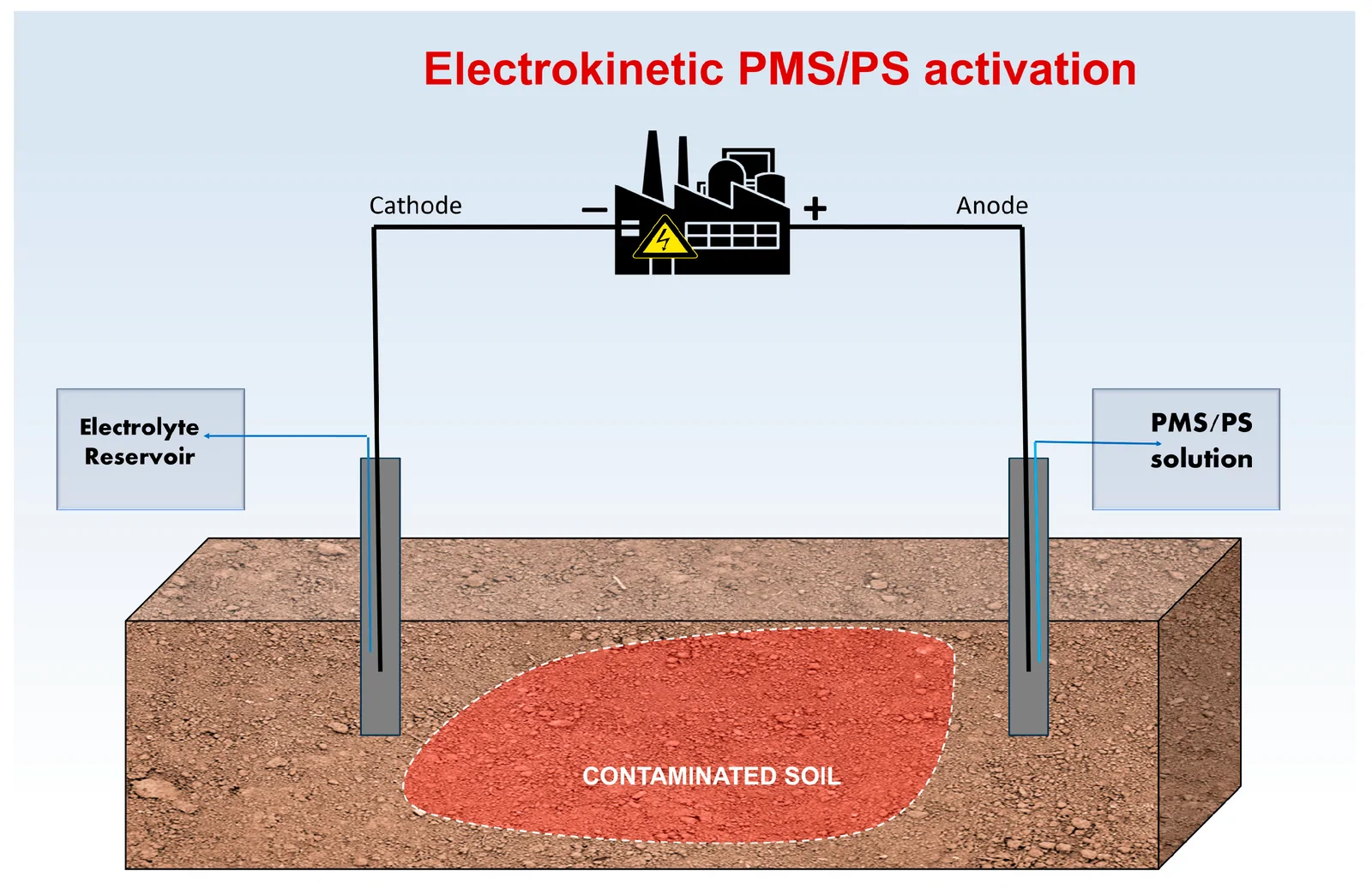
Electrokinetic PMS/PS activation.

The dispersion of oxidants into the soil can be enhanced by the so-called electrokinetic transport (see Figure 2). This process consists of the introduction of two electrodes into the ground that generates a low-voltage gradient (around 1 DCV cm^−1^) and causes a flow of charged molecules to the electrodes. PMS/PS anions (dissolved in aqueous solution) are injected directly into the soil and, by electro migration (electroosmosis), migrate to the anode. The osmotic process not only ensures a controlled flow of PMS/PS but also the mobilization of ionic/polar organic pollutants in soils with limited porosity [1,2,9,54]. The electrokinetic-facilitated transport of oxidizing agents and pollutants through the soil matrix overcomes some restrictions dictated by the permeability of the medium [55,56]. The study by Yukselen-Aksoy and Reddy [57] indeed demonstrates that the electrokinetic oxidation technique can promote PS migration even in kaolin and glacial till soil.

PS and PMS performance in combination with electrokinetics was compared in the study by Effendi et al. [55]. It was showed that PMS was able to degrade PAHs in soil more efficiently than PS. The efficiency of PMS was significantly increased when used in combination with electrokinetic techniques and cobalt and iron activators. The study found that the degradation rate progressively decreased with the increase in the molecular weight and number of rings of PAHs. This is mainly because more complex PAHs are more hydrophobic resulting in their stronger sorption and stabilization in the soil matrix. The electrokinetic/PS system in soil was also investigated in combination with zerovalent iron [56], revealing an improved activation efficiency of the oxidant. In the study by Yue et al., electrokinetics was coupled with thermal activation of PS to degrade phenanthrene in soil [58]. Thermal activation generated sulfate and hydroxyl radicals that were converted into singlet oxygen (the dominant ROS in this system) thanks to the action of electrokinetics (direct current):

(27) $$SO_{4}^{•-}+H_{2}O\to HO^{•}+SO_{4}^{2-}+H^{+}$$

(28) 2HO•+S2O82−→O21+2SO42−+2H+

All the above results suggest that the use of weak electric fields for the migration of PMS/PS and their activators is a possible valid strategy for the treatment of soil organic pollutants in situ [59,60,61].

## 5. External Activation Methods

The literature examples reported in Section 3 clearly indicate that soil components can activate PMS and PS. However, the self-activation of PMS/PS in soil is often not sufficient to achieve a satisfactory removal of pollutants [1,14]. For this reason, external activation is very often required, and this can be obtained via different methods which include heating, use of alkaline agents or transition metals, sonication and electricity sources.

### 5.1. Heat Activation

**Figure 3:**
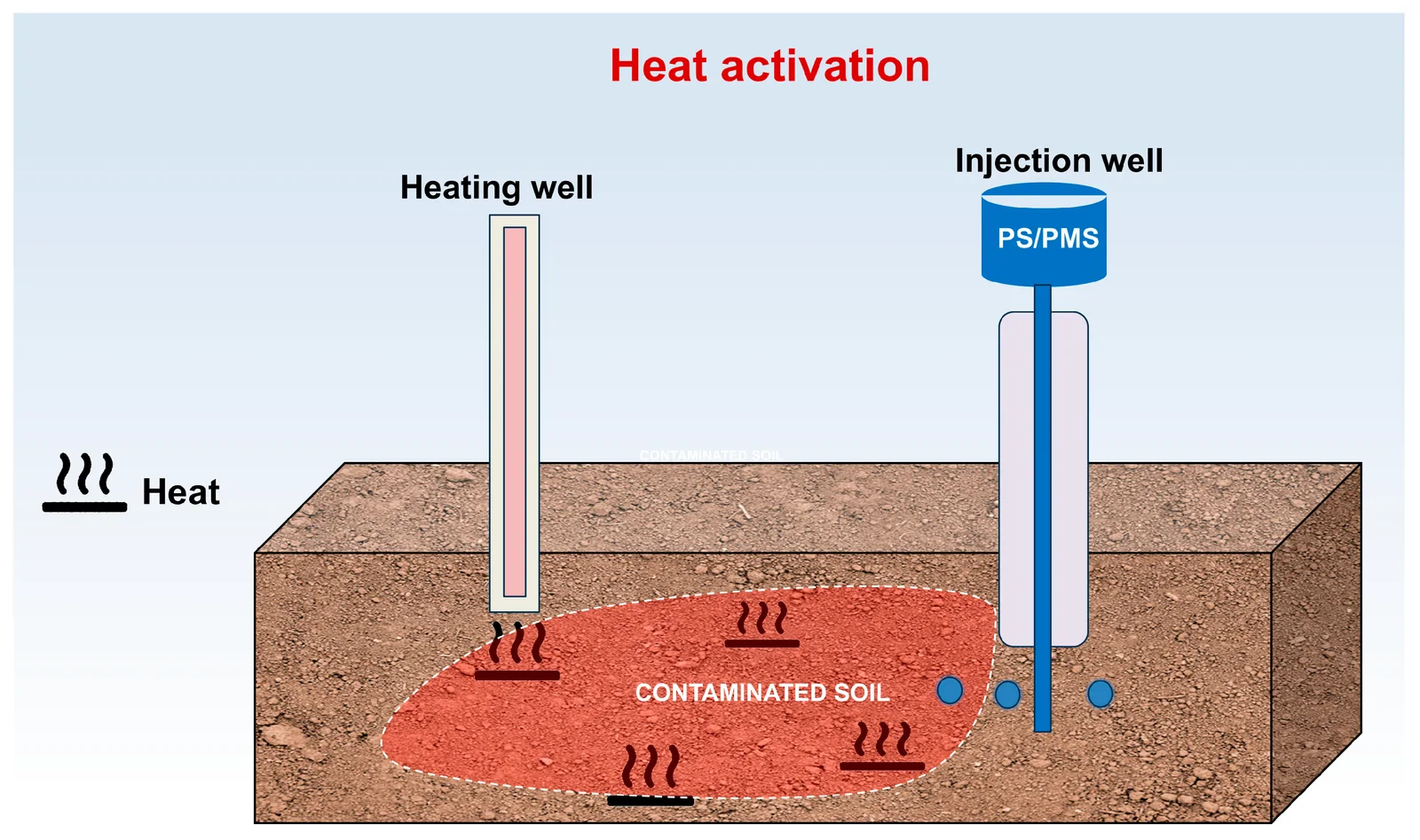
Heat activation of PMS/PS.

The activation of PS by heat (Figure 3) is currently one of the most widely used methods for many types of organic pollutants. The main limitation to this process is purely related to the associated operating costs [1,2,62]. The thermal treatment leads to the activation of PS by cleavage of the O–O bond (140 kJ mol^−1^) and the release of two sulfate radicals:

(29) $${S_{2}O_{8}}^{2-}+heat\to 2{SO}_{4}^{•-}$$

Typically, PS activation can be obtained efficiently only at temperatures above 50 °C. The energy required can vary depending on some parameters. Acidic pH can decrease energy demand, allowing the process to be triggered even at lower temperatures [1,2,9,62]. The O–O energy bond in PMS is higher (140–213.3 kJ mol^−1^), although its effective activation can occur at lower temperature with respect to PS as it possesses an asymmetric molecular structure that makes it far easier to destabilize and activate without a massive input of heat energy [63]. The results of some works on PMS/PS heat activation for soil remediation are summarized in Table 1.

One of the most practical field-scale pathways for coupling thermal activation with chemical oxidation is based on thermal conductive heating (TCH). TCH relies on borehole heater elements, electric or gas-fired, installed in vertical wells across the treatment zone, similar to those already established in thermal desorption remediation [72]. Heat is conducted outward from the heating well into the surrounding soil matrix, gradually warming it to the threshold needed for PS or PMS activation. Combining TCH with in situ chemical oxidation allows soil decontamination at lower temperatures, energy demand, and cost [67] than conventional TCH alone, since the residual heat from the thermal treatment can itself drive PMS/PS activation [72].

### 5.2. Microwave Activation

**Figure 4:**
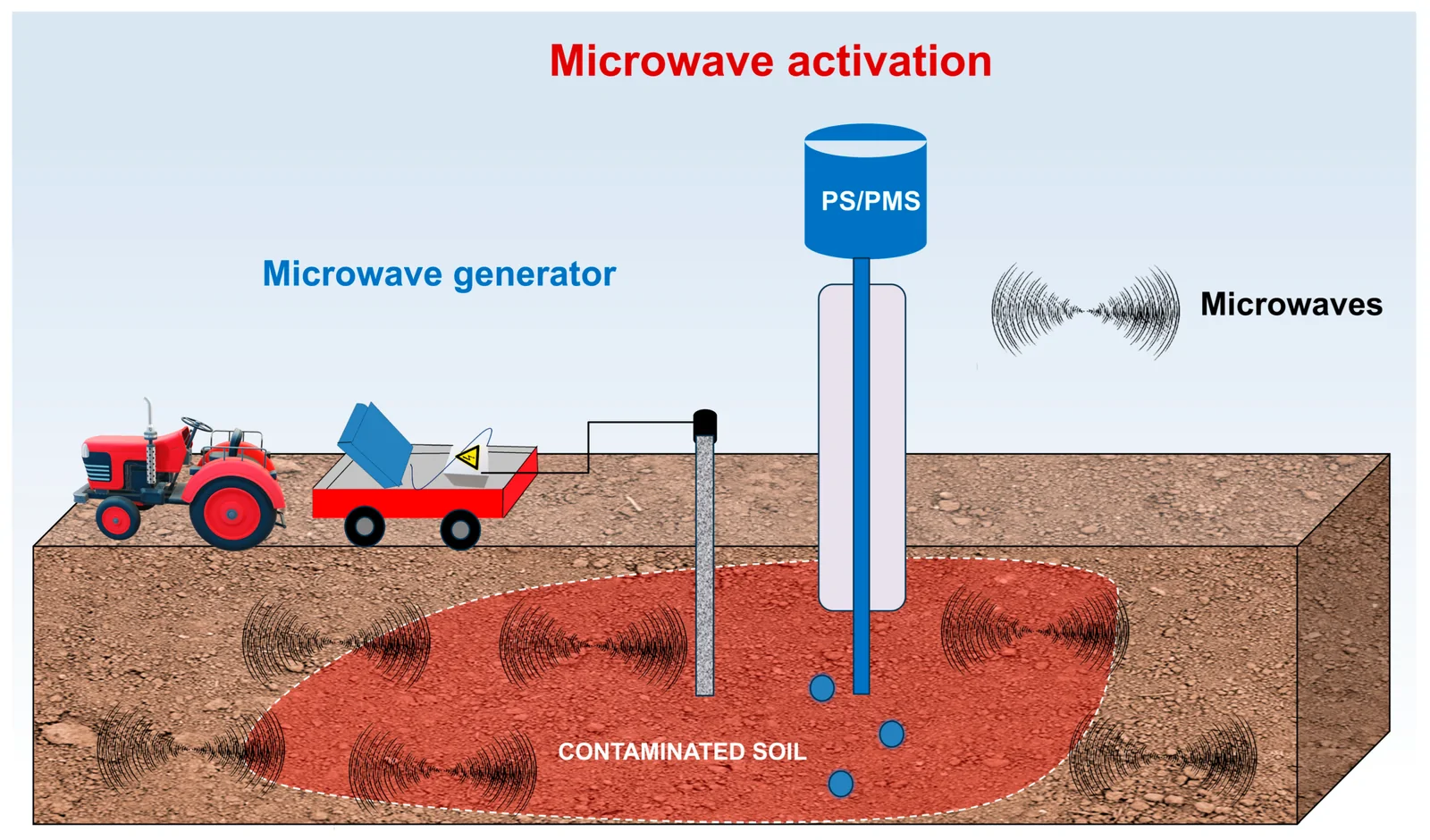
Microwave activation of PMS/PS.

Microwaves (see Figure 4) are generated by a high-frequency electromagnetic field and, compared to conventional heating processes, they can transfer energy with greater efficiency. In addition, microwaves stimulate the deformation of the PMS/PS molecular structure promoting the cleavage of the O–O bond, also by a non-thermal pathway, and hence further improve PMS/PS activation efficiency [9,73]. Furthermore, the molecular deformation induced by microwaves (MW) can even exert a degradative effect on the pollutant molecule [74].

The different oxidation capacities of PMS and PS under MW activation were explored by Shang et al. for the degradation of chlorpyrifos in soil [74]. The authors suggest that the PMS–MW system could be preferable to PS–MW for two main reasons. Firstly, PMS activation does not require elevated temperature. Secondly, the PMS–MW system is not affected by the presence of soil organic matter because its main ROS, ^1^O_2_, is strongly selective toward chlorpyrifos. Conversely, the efficacy of the PS–MW system relies on SO_4_^•−^ and HO^•^, whose availability markedly depends on the content of soil organic matter: at low soil organic matter content, SO_4_^•−^ and HO^•^ generated by MWs are partially consumed by soil organic matter itself; at high soil organic matter concentration, the efficacy of the PS–MW system increases thanks to the abundant presence of soil organic matter functional groups, such as carboxyl, hydroxyl and quinine, which are able to activate PS through an alternative pathway. Table 2 reports some examples from the literature on PMS/PS microwave activation in soil.

### 5.3. Ultrasound Activation

**Figure 5:**
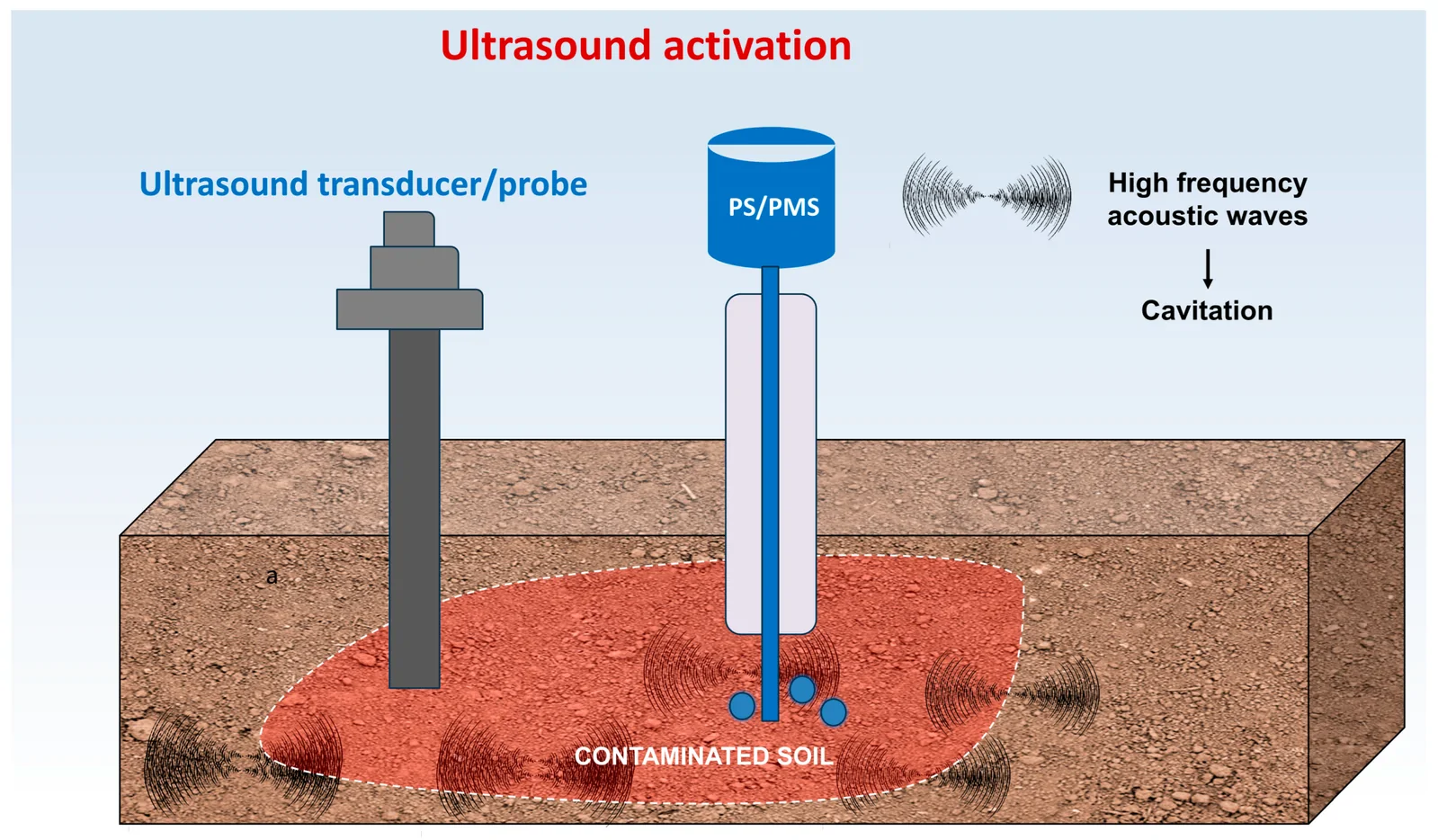
Ultrasound activation of PMS/PS.

Another source of external energy activation of PMS/PS comes from ultrasounds (Figure 5). As in the case of microwaves, ultrasounds allow a constant and homogeneous flow of energy throughout the system [81,82]. The cavitation generated by ultrasound promotes the release of hydroxyl radicals from the sonolysis of water molecules and, also, thanks to the production of heat, it activates PS and PMS [83]. As a positive side effect of cavitation, it can increase soil’s porosity and permeability. This facilitates pollutant desorption and transport of these pollutants and of oxidants within the soil [84,85].

Ultrasound-assisted PS activation was investigated, for example, by Deng et al. for treating soil contaminated with high levels of phenanthrene [86]. Ultrasound application had a dual beneficial effect on soil decontamination thanks to the generation of heat that both activated persulfate and enhanced phenanthrene desorption and dissolution. While ultrasound efficiently degrades pollutants without requiring extra persulfate (PS) activators, its primary drawback is the high operating temperature (around 70 °C) required. The results of the application of ultrasounds for the activation of PMS/PS in soil are summarized for some works in Table 3.

### 5.4. Metal Activation

**Figure 6:**
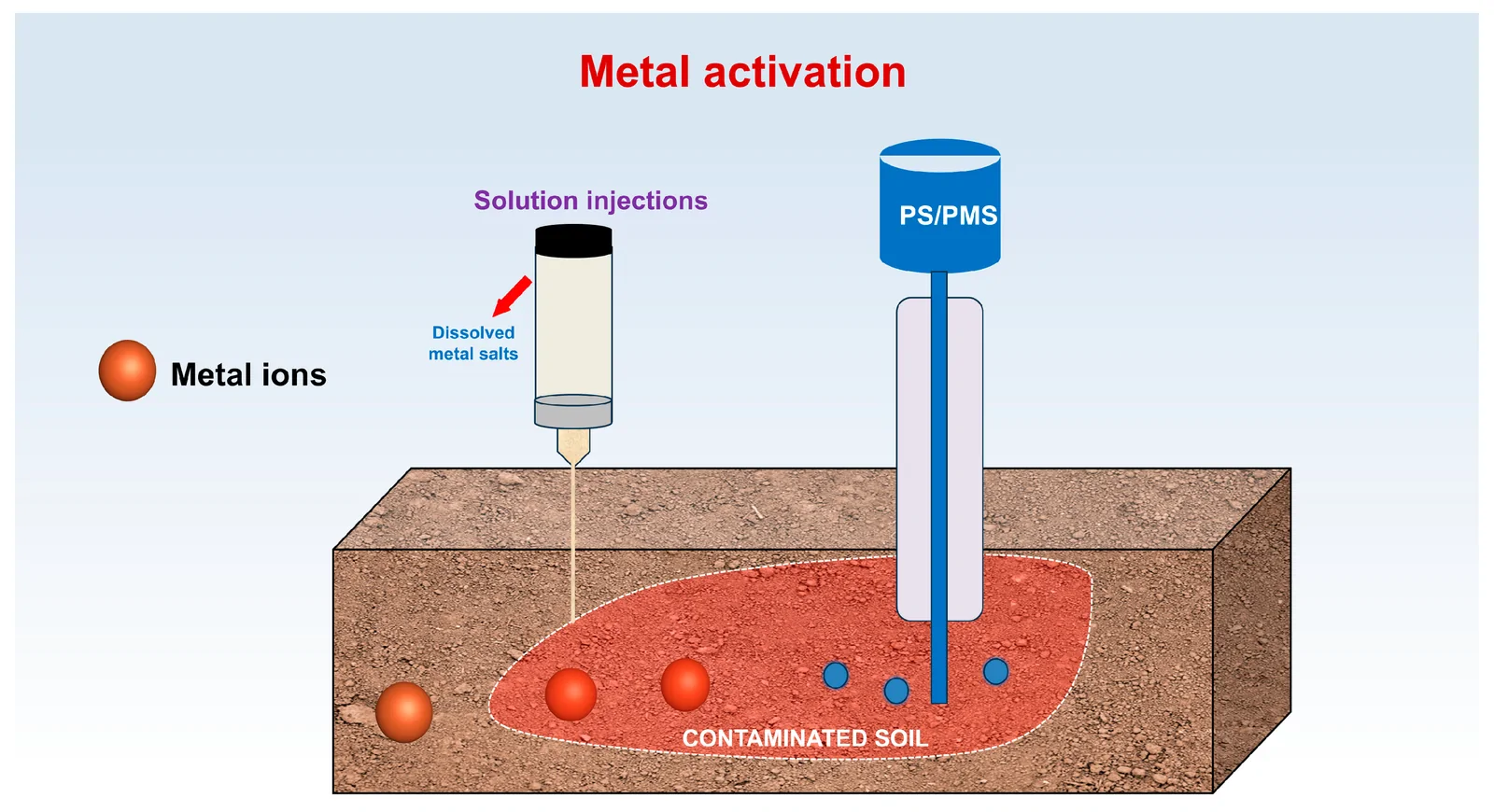
Metal activation of PMS/PS.

PMS/PS activation can also occur by transition metals such as Fe, Co, Mn, Cu, Ni, Zn and V (Figure 6). In this kind of process, the transfer of a single electron from a transition metal (M) will lead to the formation of one sulfate radical (see Equations (3) and (7)) [1,89].

#### 5.4.1. Iron-Based Activation

Iron, in the form of ferrous ion (Fe^2+^, e.g., FeSO_4_), is the most widely used metal for the activation of PMS/PS in aqueous matrices (homogeneous activation). Compared to aqueous matrices, soil requires much larger amounts of reactants (iron + PMS or PS) owing to the presence of soil organic matter that competes with the organic contaminants for the generated radicals [1,22,90]. Some examples of PS and PMS activation by iron in soil are listed in Table 4 and Table 5. The PMS/PS activation mechanism by Fe^2+^ in soil can be divided into two phases, one fast and one slow. In the fast phase, ferrous ions (Fe^2+^) react with PMS/PS, oxidizing to ferric ions (Fe^3+^) and releasing sulfate radicals [91].

(30) $${{{Fe}^{2+}+S}_{2}O_{8}}^{2-}\to {Fe}^{3+}+{SO}_{4}^{•-}+{SO}_{4}^{2-}$$

(31) $${Fe}^{2+}+{HSO}_{5}^{-}\to {Fe}^{3+}+HO^{-}+{SO}_{4}^{•-}$$

The slow phase is the regeneration of Fe^2+^ from the reduction of Fe^3+^ by oxidizing agents, such as the quinone groups of soil organic matter [1,20,22,89].

The main limitations of Fe^2+^-based activation include the need for acidic conditions (at pH ≥ 5, the iron precipitates in oxide form, escaping from the process) and the rapid consumption of Fe^2+^ which leads to a rapid exhaustion of the activator and to unwanted chain reactions with the formation of undesirable iron sludge. This problem can be avoided with the use of chelating agents that regulate the release of Fe^2+^ into the soil [1,90].

Alternatively, PMS/PS activation can be obtained by using Fe (III) salts or via heterogeneous catalysis. This latter method allows greater control of secondary reactions, thus reducing the complexity of the process. The release of ions occurs gradually because of dissolution/corrosion of the solid catalyst [22,90]. Heterogeneous activation of PMS/PS is achieved by using solid iron(II)- and iron(III)-based catalysts but also iron in zerovalent form [22]. Zerovalent iron (ZVI) is an excellent reducing agent (E_0_ = −0.44 V), readily available and non-toxic [22,89]. Contrary to PMS/PS activation method involving iron salts, the rate of sulfate radical formation in the presence of ZVI is regulated by surface reactions. The transfer of electrons from Fe^0^ to SO_4_^•−^ can occur in two ways, both requiring an acidic/weakly acidic condition. In the first case, Fe^0^ reacts in aqueous environment to form Fe^2+^ and then continues the reactions previously considered in the case of iron salts. Alternatively, Fe^0^ can react directly with PMS/PS, releasing sulfate radicals and Fe^3+^ ions. Fe^0^ can also reduce the Fe^3+^ formed, regenerating Fe^2+^ and ensuring a greater formation of radicals over time and therefore a higher yield [1,20,22,89].

(32) $${2Fe}^{0}+O_{2}+2H_{2}O\to 2{Fe}^{2+}+{4HO}^{-}$$

(33) $${Fe}^{0}+2H_{2}O\to {Fe}^{2+}+2{HO}^{-}+H_{2}$$

(34) $${Fe}^{0}+2H^{+}\to {Fe}^{2+}+H_{2}$$

(35) $${{{Fe}^{0}+2S}_{2}O_{8}}^{2-}\to \equiv Fe\left(II\right)+2{SO}_{4}^{•-}+2{SO}_{4}^{2-}$$

(36) $${Fe}^{0}+2{Fe}^{3+}\to {3Fe}^{2+}$$

ZVI is commonly produced by nucleation in a liquid solution containing a borohydrate salt which acts as a strong reducing agent for Fe^2+^ or Fe^3+^, or it can be obtained by ball milling of industrial iron powder [22]. Depending on the size of the particles, ZVI can be classified in micro-ZVI (mZVI) and nano-ZVI (nZVI). The rate of PMS/PS activation by n-ZVI is higher than that of m-ZVI as smaller particles have a greater surface area exposed to the oxidant. Although m-ZVI particles exhibit lower reactivity, they lead to the formation of iron oxides shell on their surface that may have sorptive properties toward organic contaminants [22,89,101].

In recent years there has been a progressive focus on modification techniques such as coating, supports, bimetal-modified nZVI, green synthetic nZVI, and sulfidation to improve the defects of nZVI [110]. The coating procedure stems from an interest in the improvement of the stability of nZVI within the colloidal medium, an environment in which it has low mobility due to its common adhesion to the soil mineral phase [64,111]. Obtaining a protective film on the surface of nZVI in the synthesis phase prevents particle agglomeration by electrostatic repulsion and promotes its stability. The use of surfactants can also diminish the particle size of nZVI by decreasing its charge and dipole attraction [112,113,114].

Another strategy to prevent aggregation is by using a support, such as clay materials, activated carbons, resins, montmorillonite and many others [115,116]. Qu et al. used biochar as a support for nZVI [116]. The obtained composite efficiently activated PS for removing benzo[a]pyrene from soil. Unlike conventional radical-based systems, this approach primarily uses singlet oxygen and electron transfer pathways for pollutant degradation. Support materials often also have sorbent properties, which facilitates contact between zerovalent iron and organic pollutants [110,117].

Bimetal-modification combines ZVI with other metal species with high reducing power, thus possibly increasing the reactivity of the material. However, the ecotoxicity and high price of these metals limit the application of this technique [110,118]. Green synthetic nZVI uses plant polyphenols instead of borohydride in the synthesis process [119,120,121]. Sulfide modification is a very effective process that improves the hydrophobicity of nZVI particles, allowing them to react more efficiently with organic pollutants, and reduces the formation of oxides on their surface [110,122].

As an alternative to metallic iron and iron salts, iron oxides and iron-containing minerals can also be used. Compared to the former, these have greater catalytic stability and a molecular structure that allows them to resist changes in acidity. Oxides such as Fe_2_O_3_ and FeOOH, characterized by the presence of Fe^3+^, require a first step of iron reduction to activate PMS/PS. Moreover, the dissolution of iron ores such as magnetite (Fe_3_O_4_), ferrihydrite (Fe_5_O_8_ · 4H_2_O), hematite (Fe_2_O_3_), and goethite (FeOOH) is gradually slowed down during PMS/PS activation due to the slightly acidic nature of the solutions. In addition, PMS/PS can be sorbed by the external surfaces of minerals and interact with the Fe^2+^ ions released by dissolution, with the consequent generation of radicals. These reduction and dissolution processes allow a gradual release of ferrous ions into the soil, avoiding an overproduction of radicals [56,90]. Worth mentioning is the work by Wang and co-workers for the peculiar singlet oxygen generation from the activation of PS by a Fe_3_O_4_-carbon nanotubes catalyst [123]. The authors attribute the selective generation of ^1^O_2_ to the nanoconfinement of Fe_3_O_4_ into carbon nanotubes that allows the generation of the superoxide radical (O_2_^•−^) intermediate.

(37) $$S_{2}O_{8}^{2-}+2H_{2}O\to 2SO_{4}^{2-}+O_{2}^{•-}+4H^{+}$$

(38) 2O2•−+2H2O→O21+H2O2+2HO−

(39) 2O2•−+2H+→O21+H2O2

#### 5.4.2. Other Metals

The studies conducted on the use of metal-containing catalysts other than iron for the activation of PMS/PS into soil are limited, and manganese represents one of the more promising alternatives. Like iron, manganese is non-toxic to organisms and is common in nature. Using minerals containing manganese oxides, such as pyrolusite (MnO_2_), can offer applications like those of iron ore. There are multiple studies on the efficacy of manganese oxide (MnO_2_) in activating persulfate [124,125,126] and in its combined use with ferrous materials [127,128]. It was found that in the presence of water-dissolved Fe (II), MnO_2_ gives better results than FeOOH, Fe_2_O_3_ and Fe_3_O_4_ for the activation of PS and remediation of diesel-contaminated soil. This phenomenon is primarily due to the complexation of Fe(II) on the surface of MnO_2_, creating high-density surface-bound Fe(II) that enhances PS reactivity [129]. Manganese was also investigated in the form of permanganate (MnO_4_^−^) in combination with PS for soil remediation. Despite the good oxidative efficiency of the MnO_4_^−^/PS system, it should be noted that highly ecotoxic degradation intermediates can be formed during the treatment of contaminated soils, for example by hydrocarbons [126].

Another metal tested for the generation of sulfate radicals in soil is cobalt (Co (II)). For instance, Co(II) and Fe(II) salts were used together for the activation of PMS showing good hydrocarbon removal efficiency [130]. Lyu et al. used a cobalt oxyhydroxide heterogeneous catalyst for the activation of PMS to treat oil-contaminated soil [131]. However, the negative effects of the use of cobalt or other potential toxic metals are the main reason for the limited success of this type of activation procedure. Research is currently focused on the development of heterogeneous catalysts that allow these metals to be used in less toxic and more stable forms [131,132].

### 5.5. Biochar Activation

PS and PMS activation in soil can also occur by raw biochar and the reason lies in the presence of persistent radical species (PRS) in biochar that can induce oxidant activation via electron transfer pathway. An example of this strategy is the study by Liu et al. [133] where biochar derived from lychee branch was used for the degradation of bisphenol A. In another work, Guo and co-workers prepared a biochar by pyrolyzing corn straw at 500 °C and tested it as PMS/PS activator for benzo(a)pyrene removal from soil [134]. It was found that the degradation of benzo(a)pyrene increased with the increase in biochar dosage and oxidant concentration, although excessive amounts inhibited degradation due to unnecessary SO_4_^•−^ consumption.

### 5.6. Alkaline Activation

PMS/PS activation can also be obtained under alkaline conditions. The rise in pH is generally achieved by using NaOH, KOH, or CaO [135,136]. Under such conditions, PS and PMS react with hydroxide ions to produce hydroperoxide anions (HO_2_^−^), which in turn are involved in the generation of sulfate and superoxide radicals. The latter can ultimately lead to singlet oxygen production.

(40) $$S_{2}O_{8}^{2-}+2HO^{-}\to 2{SO}_{4}^{2-}+HO_{2}^{-}+H^{+}$$

(41) $$S_{2}O_{8}^{2-}+HO_{2}^{-}\to {SO}_{4}^{2-}+{SO}_{4}^{•-}+O_{2}^{•-}+H^{+}$$

(42) $$HSO_{5}^{-}+2HO^{-}\to {SO}_{4}^{2-}+HO_{2}^{-}+H_{2}O$$

(43) $$HSO_{5}^{-}+HO_{2}^{-}\to {SO}_{4}^{•-}+O_{2}^{•-}+H_{2}O$$

(44) $$HSO_{5}^{-}+HO^{-}\to {SO}_{5}^{2-}+H_{2}O$$

(45) $$SO_{5}^{2-}+HO_{2}^{-}\to {SO}_{4}^{•-}+O_{2}^{•-}+HO^{-}$$

(46) 2O2•−+2H2O→O21+H2O2+2HO−

Owing to the strong alkalinity of the medium (pH > 12), however, the dominant ROS is often hydroxyl radical, in accordance with Equation (10) [135,136,137].

This methodology is a valid alternative to metal catalysts for the treatment of alkaline soils, where iron would not be exploitable due to the requirement for acidic conditions [135].

In addition, alkaline systems can solve (by neutralization reaction) the constant problem of acidification associated with the production of H^+^ during PS activation. This translates into lower impact on the microbial community and on the chemical–physical characteristics of the soil under consideration [138]. However, the mitigation is not unlimited and excessive H^+^ concentration may lower the pH to values at which the alkaline PS activation is interrupted. Despite the positive aspects described above of the alkaline system, the main disadvantage is related to the longer application times required for the degradation of treatment-resistant molecules, such as TPHs, in comparison to other methods, such as iron activation (see Table 6). Longer treatment results in higher monitoring costs and thus, overall, it costs similar to metal activation.

### 5.7. Mechanochemical Method

The mechanochemical method was first applied for soil remediation in 1994 [145]. The method is based on the use of high-energy crushing mechanisms, such as ball mills or modified drilling machinery, with the addition of additives like PS. The high friction and shear stress generated by collisions allow mechanical energy to be transferred across the soil material to PS where is converted into chemical energy promoting the activation process [146]. The results of mechanochemical activation of PMS/PS for some studies are summarized in Table 7.

Xu et al. recently applied the mechanochemical method for the activation of PMS and PS to remove DDTs from soil [147]. The results show that thanks to the presence of electron-rich atoms (i.e., Cl), DDTs tend to be directly oxidized by PMS and PS via non-radical pathways. PMS was found to be efficient because is more susceptible to direct oxidation than PS. Moreover, mechanical energy generated during ball milling was able to activate PMS, enhancing its oxidation ability toward DDTs. The study by Zhang et al. [148] provides interesting findings on the use of alkaline additives for improving the detoxification of chlorinated lindane by-products. It has been observed that the use of NaOH in combination with mechanochemical PS activation, markedly enhanced dechlorination rates which resulted in a complete mineralization of lindane after four hours of treatment [148]. Fan et al. analyzed the effect of organic matter on the grinding and activation process [150]. As expected, soil samples with high organic matter content exhibited lower pollutant degradation rate. The study suggests as a possible explanation the already mentioned competition of SOM for active species and/or that the presence of SOM in the sample reduces the friction between ball mill and silica component.

## 6. Side Effects of PMS/PS Treatment

In soil remediation processes that use PMS/PS, the degradation of the contaminating molecule does not necessarily coincide with a reduction in toxicity. Partial oxidation may result in the formation and accumulation of transformation products that retain toxicological activity or exhibit greater mobility than the parent compound. There are even cases in which the action of the oxidant can increase the level of toxicity, rather than eliminate it. Chu et al. report that the interaction between anthracene and PS leads to the formation of nitro-byproducts, including nitro-anthraquinone, 4-nitrophenol and 2,4-dinitrophenol derivatives. In this case, the degradation products have higher acute toxicity and mutagenic effects than the initial contaminant [153]. In another study, more than 90% of the target compound chlorpyrifos was rapidly transformed through a singlet oxygen-driven reaction (microwave/PMS system), but a substantial fraction of the parent compound was converted into 3,5,6-trichloro-2-pyridinol (TCP), a toxic and relatively mobile transformation product. The application of a second treatment allowed the degradation of TCP, this time mediated by SO_4_^•−^ and electron transfer, resulting in further dechlorination and hydroxylation and a significant reduction in the toxicity of the final products [79]. Formation of harmful nitroaromatic byproducts in the presence of NO_2_^−^ anion is also reported in the literature [41]. Thus, it is evident that it is necessary to adopt strategies capable of attacking both the contaminant and its degradation intermediates, underlining how the final decrease in the degree of soil toxicity is a more important fact rather than solely the degradation rate of the starting contaminant.

In addition to the contaminant-derived toxicity, the ecological effects of the treatment itself (introduction of PMS/PS into soil) must be taken into account as well. For example, PS oxidation of PAH-contaminated soils can temporarily suppress indigenous bacterial communities and PAH-degrading microorganisms, although microbial and enzymatic functions may partially recover during post-treatment incubation [16,17]. The impact on the microbial community of the use of oxidants is also shown in the study by Shang et al. where a chlorpyrifos-contaminated soil was treated via microwave-activated persulfate [80]. Although the acute toxicity and phytotoxicity of the treated solid phase decreased, residual oxidants in the aqueous phase retained substantial biological toxicity, highlighting a potential treatment-derived source of ecological stress [80]. Another side effect of the use of PMS/PS in soil is the salinization deriving from the massive release of ions such as sulfate. In a recent article by Wang et al. it is shown how a microbial community undergoes a reorganization in response to the salt stress post-PS treatment [154].

Soil acidification is undoubtedly one of the main causes of the reduction in the microbial communities’ activity after the consumption of PMS/PS [16]. In addition, acidification may cause the remobilization of pre-existing metals in soil. Kaur and Crimi demonstrated that the increased acidity of soil generated by PS treatment promoted the mobility of soil-bound Cd [155]. More recently, Zhang et al. reported that PMS treatment favored the transformation of Cd, Pb, and Zn from relatively stable to more active fractions in soil, resulting in increased metal availability, enhanced metal accumulation in plants, a higher potential for groundwater contamination, but also mobilization of fundamental soil resources (such as native iron), demonstrating that PMS-based oxidation may generate secondary risks beyond the removal of the target organic contaminants [156].

## 7. Cost-Effectiveness and Comparative Evaluation of Activation Methods

The PMS/PS activation methods can be compared based on three interconnected factors, (i) the energy required for activation, (ii) the sensitivity of the method to the soil properties, in particular the organic matter content and the permeability of the soil, and (iii) the secondary costs after the activation (pH maintenance, sulfate management).

Self-activation by the native soil minerals and organic matter is appealing because it involves introducing little increment in external cost other than the oxidant. The efficiencies are usually low, however, and are only practical with contaminated or high mineral content soils [4]. The simple and low-cost alkaline activation can remove aromatic hydrocarbons with good efficiency using inexpensive bases like NaOH or CaO, at ambient temperatures without needing special conditions [1]. The drawback of this is that it requires additional pH adjustment, and that it is not remarkably effective against highly hydrophobic aliphatic compounds. The metal-based activation systems, especially the ones that use zerovalent iron or iron oxides, have a good balance of cost and performance. They have low prices and high removal efficiency, but there is a risk that the presence of soil organic matter can increase the consumption of the oxidant and the production of iron sludge, which needs to be managed later [2,3]. The electrokinetic delivery of PS or PMS is in general more expensive than other methods. However, it may become economically interesting in low permeability soils, overcoming the problem of conventional injection [5]. The energy consumption in pure thermal activation is high, but when a thermal conductive heating is coupled with PMS/PS injection, overall costs can be comparable to those of conventional high temperature thermal desorption [6]. Both microwave and ultrasound activation methods yield reliable results for pollutant removal, but due to high equipment and energy costs demand, these are still not widely applied for large-scale use [7,8]. Mechanochemical treatment can be used for a few persistent organics, but the energy required for milling a large volume of soil and the subsequent requirement for improving soil quality makes this treatment less appealing [9].

Indicative cost data, although extremely limited and highly site-specific, are useful in providing a clearer perspective of the various activation methods. The cost of conventional in situ chemical oxidation using activated persulfate ranges from approximately $130 to $650 per m^3^ of treated soil, depending on the mass of the contaminants, the oxidant demand of the soil, and the number of injections needed [157,158]. Reagent costs can be 10–20% of the total project cost [158], while systems that use alkaline or iron need no continuous energy input and thus are typically the less costly systems. Thermal activation by contrast, is more expensive, even >$650 per m^3^, for difficult matrices [159]. Comparisons of the ratio of direct cost reduction and carbon emission reduction indicate that the hybrid method of thermal conductive heating–in situ chemical oxidation (TCH–ISCO) is much more competitive, achieving an average reduction of about 70% in direct costs and 80% in carbon emissions compared to conventional high-temperature thermal treatment [72]. Yet, the use of electrokinetic delivery is still developing and is estimated to be $110–$500 per m^3^ (including high costs such as electricity and electrodes), and is only economically viable if conventional injection is not possible in low permeability soils [160]. The comparison indicates that there is no one best or ideal method; each has pros and cons. In most cases, alkaline and iron-based activation provide the desired balance and can be considered as the preferred methods, while electrokinetic and hybrid thermal activation solutions are more appropriate when soil permeability or rapid removal is the main concern.

Table 8 summarizes some characteristics of each activation method.

## 8. Limitations, Conclusions and Future Outlooks

PMS/PS-based AOPs are promising processes for soil remediation. However, there are still many aspects that need further investigation. The mechanisms by which ROS are generated, interconverted in real soil, and their contribution to decontamination are not fully understood because of the physicochemical and spatial heterogeneity of soil that produces a reaction environment much more complex than that found in an aqueous system. Standard scavenging and EPR methods can provide conflicting results in the assignment of the predominant oxidants, thus requiring the development of new analytical methods (e.g., by means of isotopic labeling) under soil-relevant conditions that resolve this uncertainty. It has been seen that SOM has two possible antagonistic effects in the PMS/PS-based AOPs: at low concentrations, it can promote the formation of common oxidant radicals like SO_4_^•−^ and HO^•^ and, at high concentrations, it can completely suppress them or even shift the preferred degradation pathway from oxidative to reductive (alkyl radical mediated) or from radical to non-radical (e.g., driven by singlet oxygen, in the presence of PMS). It is still not sufficiently clear which molecular components in soil organic matter are responsible for this mechanistic bifurcation, and which quantitative thresholds induce the switch; also, a mapping of the distinctive behavior of the various soil types in this regard has not yet been carried out systematically.

The function of each soil mineral remains unclear as well. Greater efforts should be devoted to the development of predictive model connecting the identity of a mineral, the surface redox potential and the selectivity of ROS.

Although laboratory-scale tests have shown the efficacy of PMS/PS-based AOPs for the degradation of various organic contaminants in soil, still, there are some fundamental scientific shortcomings that must be addressed and a significant number of practical barriers that limit the use of this technique in field-scale-and commercial applications. For instance, due to soil heterogeneity (inconsistent permeability, SOM content, mineralogy, and moisture), the distribution of the oxidants is uneven, the contact between the oxidants and the contaminants is incomplete, resulting in an unsatisfactory removal efficiency. Low permeability zones are particularly challenging for conventional hydraulic injection because of the slow diffusion and the presence of untreated pockets due to preferential flow paths. Another important aspect to be considered is the effect of soil aging that progressively changes the physical accessibility of the contaminant and increases the importance of mass-transfer limitations. This is particularly relevant when comparing laboratory experiments performed with freshly spiked soils with weathered soils, resulting in data that overestimate the actual yield that would be achieved in real-world practice.

Energy intensive activation processes (pure thermal, microwave, ultrasound) are still too expensive to be applied on a large scale; reagent costs, multiple injection doses, and corrosion of equipment increase the project cost. Hybrid technologies, e.g., thermal conductive heating, followed with in situ chemical oxidation or electrokinetic delivery of PMS/PS, have the potential to decrease energy demand and to improve the distribution of oxidants in low-permeability soils, but they still need to be extensively pilot-scaled, the residual sulfates need to be managed according to standard procedures, and a comprehensive techno-economic and life-cycle assessment should be conducted. Specific attention should be given to the ability of added catalysts to function near neutral pH and high SOM conditions, minimizing the need for extreme pH adjustments and unnecessary doses of oxidants that are presently reducing the fertility of the soils and microbial recovery. In this regard, the use of doped/undoped biochar could be attractive because of its dual function of catalyst and, at the same time, a soil amendment agent.

The under-exploited contribution of indigenous soil microorganisms in the PMS/PS-based AOPs should also be investigated, since their response to oxidative stress (e.g., community changes), together with the possible synergistic contribution to contaminant mineralization could lead to new eco-compatible hybrid pathways.

Other limitations include the unavailability of long-term field data on ecological recovery, potential for the formation of toxic transformation products, the lack of any regulatory approach that is specifically designed for PMS/PS-based soil treatment, and the lack of validated reactive-transport models that can predict performance under realistic conditions. Current designs of large-scale systems remain very much dependent on empirical rules of thumb and site-specific trial and error, without these models and pilot data. It is therefore essential to address these issues to develop PMS/PS-based AOPs as sustainable, widely deployable remediation tools.

## Figures and Tables

**Table 1:** Heat activation of PMS/PS in soil.

Target	PMS/PS	T (°C)	Removal Efficiency	Ref.
Atrazine	PS	50 °C	68%	[62]
Ibuprofen	PS	50 °C	80%	[64]
Petroleum hydrocarbons	PS	>50 °C	73%	[65]
Cyanide	PS	60 °C	76–82%	[66]
Imidacloprid	PS	60 °C	82–97%	[67]
Hexachlorocyclohexanes	PS	55 °C	83%	[68]
DDT	PS	50 °C	92–94%	[38]
Triclosan	PS	50 °C	88%	[69]
DDT	PS	60 °C	94%	[70]
Phenanthrene (PHE)	PS	70 °C	>98%	[44]
Bisphenol A (BPA)	PMS	25 °C	100%	[27]
Pyrene	PS	80 °C	56%	[71]
PAHs	PS	69 °C	80%	[17]

**Table 2:** PS and PMS activation in soil by microwaves.

Target	PMS/PS	T (°C)	Removal Efficiency	Ref.
Atrazine	PS	80 °C	98%	[73]
Ethyl-parathion	PS	100 °C	90%	[75,76]
Phorate	PS	80 °C	87%	[77]
Terbufos	PS	80 °C	99%	[77]
Ethion	PS	80 °C	91%	[77]
Parathion	PS	80 °C	90%	[77]
Chlorpyrifos	PS/PMS	80 °C	85%/94%	[74]
Pyrene	PS	60 °C	92%	[78]
Pyrene	PS	80 °C	85%	[71]
Chlorpyrifos	PMS	60 °C	>90%	[79]
Imidacloprid	PMS	80 °C	64–91%	[80]
Imidacloprid	PS	80 °C	100%	[80]
Chlorpyrifos	PMS	60 °C	>90%	[35]

**Table 3:** PS and PMS activation in soil by ultrasounds.

Target	PMS/PS	Frequency (kHz)	Power (W)	Removal Efficiency	Ref.
Petroleum hydrocarbons	PS	-	200	56–82%	[87]
Petroleum hydrocarbons	PS		200	78%	[65]
PFAS	PS	20–43	250–500	62–71%	[88]
Phenanthrene	PS	20	90	79–96%	[86]

**Table 4:** Iron (except zerovalent) activation of PS and PMS in soil.

Target	PMS/PS	Catalyst	T (°C)	Removal Efficiency	Ref.
Polycyclic aromatic hydrocarbons (PAH)	PS	Fe^2+^ + citric acid + surfactant IGEPAL CA-720	-	81%	[16]
Triclosan	PS	Fe^2+^	23 °C	80%	[92]
Ibuprofen	PS	Fe^3+^ + gallic acid	25 °C	81%	[93]
Naphthalene	PS	Fe^2+^	25 °C	62%	[94]
Ibuprofen	PS	Fe^2+^	-	72%	[95]
Pyrene	PMS	amorphous porous Fe	25 °C	98%	[96]
Sulfamethoxazole	PS	Fe oxides	25 °C	71%	[97]
Bensulfuron methyl	PMS	Fe_2_O_3_/Fe_3_O_4_@C	-	64%	[98]
Quinclorac	PMS	FeS_2_@ Biochar	25 °C	91%	[99]

**Table 5:** Zerovalent iron activation of PS and PMS in soil.

Target	PMS/PS	Catalyst	Removal Efficiency	Ref.
Para-chlorobenzene	PS	ZVI	89%	[100]
Polycyclic aromatic hydrocarbons	PS	nZVI	82%	[101]
BDE209	PS	nZVI + kaolin	89%	[102]
1,2-dichlorobenzene	PS	nZVI	97%	[103]
Neonicotinoids	PS	nZVI	69–89%	[104]
Decabromodiphenyl ether	PS	Modified nZVI	81%	[105]
Pyrene	PS	N-doped-biochar supported nZVI	95–99%	[106]
Petroleum hydrocarbons	PS	nZVI + Electrokinetic	40%	[56]
Polycyclic aromatic hydrocarbons	PS	Activated Carbon Supported nZVI	61–91%	[107]
Polycyclic aromatic hydrocarbons (PAH)	PS	Fe-Cu + Activated carbons	82%	[108]
Polycyclic aromatic hydrocarbons (PAH)	PS	Fe-Ni + Activated carbons	96%	[109]

**Table 6:** Alkaline activation of PS and PMS in soil.

Target Contaminant	PMS/PS	Activation Treatment	Removal Efficiency	Treatment Duration	Ref.
Diesel/TPH	PS	NaOH/CaO	30%	7 d	[139]
Aged diesel/TPH	PS	NaOH	60–77%	56 d	[135]
Naphthalenes + phenanthrenes	PS	NaOH	80%	4 d	[135]
TPH	PS	NaOH	55%	56 d	[135]
PAHs + TPH	PS	NaOH + surfactant	70%	25 d	[140]
HCHs/chlorinated organic compounds	PS	NaOH	70–96%	21 d	[141]
diesel weathered/NAPL	PS	NaOH	80%	-	[142]
Pentachlorophenol	PS	NaOH/CaO	99.4%	3 d	[143]
Diesel/TPH	PS	CaO	86%	7 d	[144]

**Table 7:** Mechanochemical activation of PS and PMS in soil.

Target	PMS/PS	Milling Speed (rpm)	Removal Efficiency	Ref.
PAHs	PS	500	82.5%	[146]
DDTs	PMS	500	97%	[147]
Lindane	PS + NaOH	550	100%	[148]
Lindane	PS + CaO	550	90%	[148]
Lindane	PS + Fe	550	75%	[148]
UV-328	PS	350	92%	[149]
UV-328	PMS	350	94%	[149]
phenanthrene	PMS	400	>98%	[150]
HCB	PS/CaO	350	85%	[151]
tris(1-chloro-2-propanyl) phosphate (TCPP)	PS/ZVI	400	>83%	[152]

**Table 8:** Comparison of activation methods for PMS/PS soil remediation.

Method	Cost	Advantage	Practical Limitation	Ref.
Alkaline	Low	Simple, ambient conditions	pH adjustment needed	[1]
Iron/ZVI	Low to moderate	Inexpensive catalysts, high efficiency	Sludge and excess oxidant demand	[2,3]
Self-activation	Very low	No external activator	Limited on heavily polluted soils	[4]
Electrokinetic	Moderate	Works in low-permeability soils	Electrode installation costs	[5]
TCH–ISCO hybrid	Moderate	Substantial energy savings vs. pure thermal	Still requires heat input	[6]
Microwave	High	Fast and efficient heating	Equipment and energy cost	[7]
Ultrasound	High	Improves desorption	High power demand	[8]
Mechanochemical	High	Minimal liquid reagents	Milling energy and soil recovery	[9]

## Data Availability

No new data were created or analyzed in this study. Data sharing is not applicable to this article.
